# Efficacy of various prescribed vitamin D supplementation regimens on 25-hydroxyvitamin D serum levels in long-term care

**DOI:** 10.1017/S1368980021001609

**Published:** 2022-01

**Authors:** Ronna N Robbins, Monica Serra, Nalini Ranjit, Deanna M Hoelscher, Sara J Sweitzer, Margaret E Briley

**Affiliations:** 1 The University of Texas at Austin, Nutritional Sciences School of Human Ecology, 200 W. 24th Street, GEA 331, Austin, TX 78712, USA; 2 Division of Geriatrics, Gerontology & Palliative Medicine and the Sam & Ann Barshop Institute for Longevity & Aging Studies, Department of Medicine, UT Health San Antonio, San Antonio, TX, USA; 3 San Antonio GRECC, South Texas Veterans Health Care System, San Antonio, TX, USA; 4 Michael & Susan Dell Center for Healthy Living, The University of Texas Health Science Center at Houston (UTHealth), School of Public Health Austin Regional Campus, Austin, TX, USA

**Keywords:** Vitamin D, Supplementation, 25-Hydroxyvitamin D, Long-term care, Older adults, Skilled nursing

## Abstract

**Objective:**

The aims of this study were to examine the efficacy among various vitamin D supplementation regimens on serum 25-hydroxyvitamin D (25(OH)D) concentrations and determine the minimal dose rate required to achieve sufficient serum concentrations (≥75 nmol/l) among older adults in long-term care (LTC).

**Design:**

A 1-year medical history was abstracted from medical records, and a one-time blood draw to measure serum 25(OH)D concentrations was obtained. Individuals were stratified into vitamin D-supplemented and non-supplemented groups. The supplemented group was further categorised into four treatment forms: single-ingredient vitamin D_2or3_, multivitamin, Ca with vitamin D or combination of the three, and by daily prescribed doses: 0–9·9, 10–19·9, 20–49·9, 50–99·9 and >100 μg/d.

**Setting:**

Five LTC communities in Austin, Texas.

**Participants:**

One hundred seventy-three older (≥65 years) adults.

**Results:**

Of the participants, 62% received a vitamin D supplement and 55% had insufficient (≤75 nmol/l) 25(OH)D serum concentrations. Individuals receiving single-ingredient vitamin D_2or3_ supplementation received the highest daily vitamin D mean dose (72·5 μg/d), while combination of forms was the most frequent treatment (44%) with the highest mean serum concentration (108 nmol/l). All supplementation doses were successful at reaching sufficient serum concentrations, except those<20 μg/d. Using a prediction model, it was observed that 0·025 μg/d of vitamin D supplementation resulted in a 0·008 nmol/l increase in serum 25(OH)D concentrations.

**Conclusions:**

Based on the predictive equation, results suggest that supplementation of 37·5 μg/d of vitamin D_2or3_ or combination of vitamin D is most likely to achieve sufficient serum 25(OH)D concentrations in older adults in LTC.

The classical function of vitamin D in Ca and phosphate homoeostasis and bone metabolism has long been recognised. Over the past decades, a more expansive role of vitamin D in non-skeletal physiological processes has emerged^(1,2)^. Research shows that insufficient serum vitamin D concentrations are associated with an increased rate of respiratory tract infection, influenza and other infectious diseases, along with several chronic health conditions, including dementia, depression, CVD and cancer^(3–7)^. As the world deals with the health and economic burden of the corona virus disease of 2019 (COVID-19) pandemic, insufficient vitamin D concentrations have emerged as a possible risk factor for severe acute respiratory syndrome coronavirus (SARS-CoV-1) infection, the virus that causes COVID-19. As a result, the interest in vitamin D supplementation to reduce the rate of infection, lessen severe illness and/or accelerate recovery has surfaced^(5–7)^.

Older adults, especially those in long-term care (LTC), experience high rate of vitamin D insufficiency (40–100%) due to inadequate sunlight exposure, medication interactions and limited dietary sources^(1,4,6,8,9)^. LTC older adults are also among the most vulnerable populations for SARS-CoV-1 outbreaks and at the greatest risk for severe illness and morality from COVID-19^(10,11)^. Correcting insufficient vitamin D concentrations in these individuals is critical secondary to vitamin D’s role in regulating innate and adaptative immunity, and potential to reduce the risk of viral infection, progression and severity^(5,6)^. Since LTC residents are often exposed to limited sunlight exposure, oral nutrition becomes an essential route for intake of vitamin D; hence, supplementation is recommended to maintain optimal serum 25-hydroxyvitamin D (25(OH)D) concentrations^(12,13)^.

The National Academy of Medicine, formerly known as Institute of Medicine, set the dietary reference intake for vitamin D in older adults (70+ years) at 20 μg/d, which is sufficient to reach serum 25(OH)D concentration of ≥50 nmol/l (20 ng/ml) and maintain bone health^(12–15)^. However, a growing body of evidence suggests that 20 μg/d will not raise serum 25(OH)D concentrations above the Endocrine’s Society concentration recommended to achieve sufficiency (75 nmol/l (30 ng/ml))^(12)^. Thus, the dietary reference intake may not be protective of non-skeletal health conditions including COVID-19^(6,12,16)^. Even with general agreement among health organisations and experts for universal vitamin D supplementation in older adults (≥65 years), the recommended supplementation dose rate and 25(OH)D target serum concentrations remain controversial^(12,16,17)^. Health organisations and experts suggest a variety of supplementation dose rates ranging from 25 to 100 μg/d to achieve sufficient serum 25(OH)D concentrations^(18–21)^.

Despite the known health consequences, environmental and physiological risk factors, and recommendations from experts for universal blood screening and supplementation, vitamin D insufficiency is routinely not diagnosed and/or undertreated within the LTC population^(1,8,9,16,17,22)^. Several factors contribute to poor testing and treatment, including limited coverage of 25(OH)D blood test by Medicaid/Medicare and lack of systematic supplementation by practitioners in LTC, resulting from a perception that supplementation and/or correcting for insufficiency is not considered a health priority^(17,23–25)^.

The objectives of this study were to determine the prevalence of vitamin D supplementation and examine the efficacy among various vitamin D supplementation regimens on serum 25-hydroxyvitamin D concentration in older adults living in a LTC community. Additionally, we aimed to determine the minimal vitamin D supplementation dose rate required to achieve sufficient serum 25(OH)D concentration (≥75 nmol/l) in these LTC patients.

## Methods

### Participants

For this cross-sectional study, older adults from five LTC communities in the metropolitan area of Austin, Texas, were recruited to participate. To be eligible, participants had to be ≥65 years and reside within skilled nursing or assisted living units. There were no exclusion criteria for 25(OH)D serum concentrations or vitamin–mineral supplementation. Written consent was obtained from medical power of attorney for all participants, along with verbal assent from individuals without cognitive impairment (assessed by each patient’s nursing staff or Social Worker).

### Data collection

A 1-year medical history was collected from on-site electronic medical records using double-blinded protocols^(26,27)^. Data abstracted from electronic medical records included demographics (age, sex, race and level of care), lifestyle factors (alcohol and tobacco use) and medical history (weight, height, diagnoses, medications, number of infections, falls and hospitalisations). Race was categorised as Caucasian or non-Caucasian based on the U.S Census Bureau’s 2013–2017 American Community Survey^(28)^. Race was included as a covariate to account for any potential difference in serum concentrations secondary to skin pigmentation and UV-B (sun) exposure. Height and weight from the electronic medical records were used to calculate BMI as kg/m^2^ and then categorised into the Center for Disease Control Adult standard weight status categories: underweight: <18·5 kg/m^2^; healthy weight: 18·5–24·9 kg/m^2^; overweight: 25·0–29·9 kg/m^2^ and obese: ≥30·0 kg/m^2(29)^. The dosage, start and/or discontinue date for all medications were collected and then categorised according to the Food and Drug Administration’s U.S. Pharmacopeia Therapeutic Category and Pharmacologic Classification Guidelines (e.g. antidepressants, diuretics and bisphosphates)^(30)^. Medications that inhibit or induce cytochrome P450 25-hydroxylase enzyme activity, which is responsible for converting ergo- and cholecalciferol (dietary sources of vitamin D) to the circulating metabolite 25(OH)D, were further categorised as having a drug–vitamin D interaction and used as a covariate in statistical analyses^(31)^.

Vitamin–mineral supplementation along with dosage, start and/or discontinue date was also collected from the medical record. Vitamin D supplementation was defined as a supplement containing ≥5 μg/d of vitamin D_2_or_3_, which included single-ingredient vitamin D supplement, Ca with vitamin D and multivitamins. To determine the specific amount of vitamin D provided by multivitamins, each LTC community provided their house multivitamin formulary.

### Nutritional analysis

Each community provided their spring/summer 2018 cycle menus along with the corresponding nutritional analyses and serving sizes. The nutritional analyses did not include vitamin D, so a trained LTC-Registered Dietitian Nutritionist used the USDA Nutrient Composition Database to calculate the estimated daily average of vitamin D (μg) provided in meals^(32)^. Nutritional analyses were conducted using generic recipes and community-specific serving sizes. To ensure the calculated analysis was credible, the macro- and micronutrients of the calculated analyses were compared with the nutritional analysis provided by each LTC community. The amount of vitamin D provided from oral nutritional supplementation (i.e. energy/protein shakes) was documented and included in the daily vitamin D meal total for each participant.

### Measurements/serum analysis

Despite the expected time spent outdoors and accompanying sun exposure to be minimal for LTC patients, fasting venous blood draws were obtained from all participants during the summer of 2018, when serum 25(OH)D concentrations were predicted to be the highest (average high temperature of 35·1ºC (95·2ºF) in Austin, Texas)^(12,33,34)^. The Endocrine Society clinical practice guidelines were used to define serum 25(OH)D concentrations as sufficient (≥75 nmol/l) and insufficient (<75 nmol/l)^(12)^. To ensure each LTC community maintained regulatory compliance with the Texas Department of Health and Human Services and the Center for Medicare and Medicaid Services, a mobile diagnostic laboratory and phlebotomy company that was both College of American Pathologists Accredited and CLIA-88 certified (Clinical Laboratory Improvement Amendments) were contracted to collect blood samples. All test procedures followed the Clinical Laboratory Standards Institute Evaluation Protocols. An Access 25(OH)D vitamin D total chemiluminescent immunoassay was used to measure serum 25(OH)D concentrations using the Access2 Immunoassay System (Beckman Coulter). The Beckman Coulter Access 25(OH)D vitamin D Total assay is standardised and traceable to the gold standard 25(OH)D vitamin D Reference Measurement Procedure (RMP) from Ghent University (Ghent, Belgium)^(35)^. Using Passing-Bablok regression and Spearman correlation, a measurement procedure comparison evaluated serum samples (*n* 110) with Access 25(OH)D vitamin D Total assay (ng/ml) on the Access2 System and an isotope-dilution-LC-tandem-MS/MS 25(OH)D vitamin D (RMP by Ghent University) and produced the following results: *R*-value 0·95 (intercept-2·87, 95 % CI −5·44, −0·88, slope 1·01 (0·94, 1·10))^(35)^. Beckman Coulter reports the Access 25(OH)D vitamin D assay to have a total CV≤10%, which meets the vitamin D Standardization Program criteria^(35)^. Supporting this claim is an independent 2017 study by Madenci *et al.*, which evaluated the 25(OH)D vitamin D Total assay (ng/ml) on the Access2 System analytical performance and found a CV% of 8·1 and 7·7% for low and high concentrations, respectively^(36)^. Per manufactures specification and laboratory policy, the Access 25(OH) vitamin D Total assay undergoes quantitative assay calibration every 28 d using assay calibrators, which are traceable to the Joint Committee for Traceability in Laboratory Medicine-approved isotope-dilution-LC-tandem-MS/MS and RMP developed at Ghent University. The calibrator’s RMP is further traceable to the National Institute of Standards and Technology standard reference material 2972^(33)^. To validate the accuracy of analysis, all critical concentrations were automatically re-analysed, a delta check was conducted on all specimens and a significant deviation (*P*<0·01) prompted a re-test.

Individuals were stratified into groups based on whether they received vitamin D supplementation or were non-supplemented. The vitamin D-supplemented group was further categorised based on the following treatment forms: single-ingredient vitamin D (vitamin D_2or3_), Ca with vitamin D, multivitamin and combination of the three treatments. The total study population was categorised by supplementation dose rate prescribed per day: 0–9·9, 10–19·9, 20–49·9, 50–99·9 and >100 μg/d. The 0–9·9 μg/d supplemented group included those not receiving any supplementation. The dose range of 10–19·9 μg/d was determined based on multiple studies that showed supplementation <20 μg/d does not raise serum 25(OH)D concentrations above 75 nmol/l^(20–22,37)^. The dose range of 20–49·9 μg/d was determined by the dietary reference intake recommendation of 20 μg/d and several randomised control trials that did not detect serum differences in supplementation dose rates between 20 and 50 μg/d^(15,21,37–40)^. The dose range of 50–99·9 μg/d was determined based on limited prior data available on serum concentration effects for these doses. Finally, the dose range >100 μg/d was selected based on randomised control trials showing supplementation of 1000 μg/d or higher resulted in serum concentrations ≥75 nmol/l^(12,21,41)^.

### Statistical analysis

Statistical analyses were performed using Stata v16 (StataCorp.). Descriptive analyses summarised population characteristics; 25(OH)D serum concentrations; supplementation treatment types and dose rates; and the prevalence of vitamin D sufficiency (≥75 nmol/l) and insufficiency (<75 nmol/l). Continuous variables are presented as mean with their standard errors and as percentages for categorical variables. Depending on variable *t* test, *χ*^2^ or ANOVA determined mean differences in serum 25(OH)D concentrations. Factorial ANCOVA compared serum 25(OH)D concentrations across different dietary supplementation treatment types and dose ranges. Covariates in ANCOVA included: vitamin D provided in meals (μg/d), years living in the LTC community, age, race, sex, BMI, diagnosis of renal and liver disease, and prescribed medications with a drug–vitamin D interaction. Bonferroni-type adjustment corrected for multiple comparisons with an adjusted significant *P*-value of *P*≤0·005. Linear regression determined the minimal dose of vitamin D supplementation required to achieve sufficient serum concentrations (≥75 nmol/l). Multiple logistic regression was used to determine significant predictors of sufficient serum 25(OH)D concentrations among the various treatment regimens within supplementation dose rates and treatment forms while controlling for the above covariates. *P*-values of <0·05 were set as the threshold for statistical significance for the ANCOVA models.

## Results

A total of 180 participants were recruited; however, four refused blood draws, and three died during the study period. Therefore, analyses were performed on 173 participants with complete data. Participants were older (age: 83±0·82 years), and the majority were Caucasian (89%), women (61%) and overweight (BMI: 26±0·43 kg/m^2^). Thirty-eight percentage were not prescribed a vitamin D supplement, and 55% of participants had insufficient 25(OH)D (<75 nmol/l). Participants were prescribed an average of 11 (range 1–22) medications per day (including on average 2 vitamin–mineral supplements), with 52% receiving at least one medication with a drug–vitamin D interaction. The calculated estimated daily average intake of vitamin D provided in meals (which included fortified foods) was 5 μg/d. As seen in Table 1, the mean serum dose rate of vitamin D supplementation and 25(OH)D concentrations did not differ between demographic characteristic groups (sex, race and level of care).

**Table 1:** Population characteristics: mean supplementation dose rates and serum 25(OH)D concentrations

	*n*, %	Dietary supplement intake dose rate (μg/d) (mean, se)†	*P*-value‡	Serum 25(OH)D (nmol/l) (mean, se)†	*P*-value‡	Insufficient (<75 nmol/l) (%)	*P*-value‡
Sex							
Female	107, 61	30, 6		83, 4		53	
Male	66, 39	30, 5	0·957	80, 3	0·731	58	0·524
Race							
Caucasian	159, 89	31, 6		83, 3		55	
Non-Caucasian	19, 11	21, 4	0·460	70, 7	0·216	53	0·813
Level of care							
AL	62, 35	33, 8		89, 3		48	
SNF	111, 65	28, 4	0·501	77, 3	0·063	59	0·181
Medical diagnosis							
Liver disease*	8, 5	21, 6	0·629	55, 15	0·061	87	0·060
Renal disease*	55, 32	38, 6	0·213	83, 4	0·942	44	0·024
Medication interaction‖	90, 52	32, 7	0·555	75, 6	0·155	45	0·563
Total population	173, 100	30, 4		80, 3		55	
Vit D supplemented	108, 62	50, 6		94, 4		42	
Non-supplemented	65, 38	0.2, 0.1	<0·001	60, 3	<0·001	81	<0·001
Supplementation treatment forms§							
Vit D_2or3_	29, 27	73 14		92, 6		35	
Ca + D	14, 13	13, 2		73, 6		50	
MVI	17, 16	12, 1		71, 5		77	
Combo	48, 44	58, 8	<0·001	108, 8	<0·001	30	<0·001
Supplement dose range (IU/d)§							
0–399	70, 41	0.2, 0·1		63, 3		79	
400–799	27, 45	11, 0·4		73, 5		59	
800–1999	30, 17	27, 2		88, 5		43	
2000–3999	30, 17	58 1		104, 6		27	
>4000	16, 9	152, 23	<0·001	133, 19·5	<0·001	19	<0·001

AL, assisted living; SNF, skilled nursing facility; Vitamin D_2or3_, supplement containing either vitamin D_2_ or D_3_; MVI, multivitamin; Ca + D, Ca + vitamin D; Combo, combination of vitamin.

* Compared with not having diagnosis of liver or renal.

† Supplementation continuous variable reported as means with their standard errors.

‡ *P*-value determined by *t*-text, *χ*^2^ or ANCOVA depending on variable.

§ Percentages within each category.

‖ Compared with medication without drug–vitamin D interaction.

ANCOVA Covariates: age, BMI, sex, years living in community, diagnosis of liver or renal disease, vitamin D provided in meals and oral supplements, and medication with a drug–vitamin D interaction.

### Mean comparisons between different supplementation treatment forms

Only four individuals received vitamin D_2_ alone, so vitamins D_2_ and D_3_ were combined into one treatment category (vitamin D_2or3_). As seen in Table 1, the most commonly provided vitamin D supplement was a combination (44%), with the least being coming from a Ca + vitamin D supplement (16%). The mean dose rate of those supplemented was 50±6 μg/d (range 5–375 μg/d), with the highest dose provided by the vitamin D_2or3_, followed by the combination treatment forms. Mean serum 25(OH)D concentration was 94±4 nmol/l. As expected, when compared with non-supplemented individuals, those supplemented had a higher vitamin D intake (50 *v*. 0·2 μg/d, *P*<0·001) and serum 25(OH)D concentrations (94±4 *v*. 60±3 nmol/l, *P*<0·001), on average. Insufficient serum concentrations were observed in 42% of those supplemented compared with 81% non-supplemented (*P*<0·001).

Table 1 also demonstrates that mean supplementation dose rates, serum 25(OH)D concentrations and prevalence of insufficient serum concentrations differed significantly across all four forms of vitamin D supplementation treatment types (all *P*<0·001), with those receiving vitamin D from a D_2or3_ or combination sources achieving the highest serum concentrations. Serum 25(OH)D also significantly increased as supplementation dose increased (all *P*’s<0·001). As expected, a higher prevalence of serum vitamin D insufficiency was observed in those in the 0–9·9 μg/d group compared with the >100 μg/d group (79 *v*. 19%; *P*<0·001).

Table 2 shows pairwise comparisons of serum 25(OH)D concentrations across the different supplementation treatment forms. It was observed that compared with the non-supplemented group, those in the vitamin D_2or3_ and combination groups had approximately 30–40 nmol/l higher serum 25(OH)D concentrations (*P*’s<0·001). Further, consuming a combination of supplements also results in approximately 30 nmol/l higher serum 25(OH)D concentrations than that observed in the multivitamin group (*P*= 0·003). Table 2 also shows pairwise comparisons of mean serum 25(OH)D concentrations across the different supplementation dose ranges. In general, it was observed that serum concentration significantly increased as supplementation dose increased.

**Table 2.** Pairwise comparison of serum 25(OH)D concentrations across supplementation treatment forms and dose ranges

	Mean difference* (nmol/l)	Bonferroni 95% CI	*P*-value†
Supplement treatment type			
Vit D_2or3_ *v*. No D supplement	30·3	7·5, 53·3	0·001
Ca + Vit D *v*. No D supplement	22·0	−12·0, 48·0	0·070
MVI *v*. No D supplement	8·0	−20·0, 35·6	1·000
Combo *v*. No D supplement	39·5	27·8, 66·5	0·001
Ca + Vit D *v*. Vit D_2or3_	−8·3	−45·6, 20·5	1·000
MVI *v*. Vit D_2or3_	−22·3	−54·0, 8·0	0·130
Combo *v*. Vit D_2or3_	9·3	−7·5, 40·5	1·000
MVI *v*. Ca + Vit D	−14·0	−47·0, 26·5	1·000
Combo *v*. Ca + Vit D	17·5	−1·8, 60·0	0·410
Combo *v*. MVI	31·5	10·8, 68·3	0·003
Supplement dose range (μg/d)			
10–19·9 *v*. 0–9·9	11·0	−11·0, 33·0	1·000
20–49·9 *v*. 0–9·9	24·5	3·0, 46·0	0·001
50–99·9 *v*. 0–9·9	42·5	21·3, 64·0	0·001
>100 *v*. 0–9·9	69·0	42·3, 69·0	0·001
20–49·9 *v*. 10–19·9	13·3	−12·8, 39·0	1·000
50–99·9 *v*. 10–19·9	31·5	5·5, 57·0	0·005
>100 *v*. 10–19·9	58·3	27·5, 89·0	0·001
50–99·9 *v*. 20–49·9	18·3	−7·25, 43·5	0·430
>100*v*. 20–49·9	44·8	14·5, 75	0·001
>100 *v*. 50–99·9	26·5	−3·5, 56·8	0·130

Vitamin D_2or3_, supplement containing either vitamin D_2_ or D_3_; MVI, multivitamin; Ca + Vit D, Ca + vitamin D; Combo, combination of vitamin D supplementation types.

* Difference of means between pairs (nmol/l).

† *P*-value determined by ANCOVA with Bonferroni adjustments.

Adjusted *P*-value significant at *P*<0·005.

Covariates: age, BMI, sex, years living in community, diagnosis of liver or renal disease, vitamin D provided in meals, and medication that interferes with vitamin D metabolism.

Linear regression was performed to determine the predicted vitamin D supplementation dose rate required to achieve sufficient serum 25(OH)D concentration. The model (*R*^2^=0·31 (95 % CI 0·03, 0·005), *P*<0·001) explained 36% of the variability between serum concentrations and supplementation dose rate. The equation predicting minimal supplementation dose rate required to achieve sufficient serum 25(OH)D concentrations of ≥75 nmol/l is:

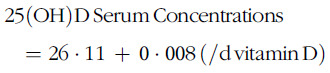




Thus, 1 IU/d of vitamin D supplementation resulted in a 0·008 nmol/l increase in serum 25(OH)D concentrations. Using this predictive equation, a supplementation dose rate of 37·5 μg/d would be required to achieve sufficient serum 25(OH)D concentration of 75 nmol/l.

Of the treatment forms, only vitamin D_2or3_ (OR: 9·0 (95 % CI 3·1, 25·7), *P*<0·001) and combination of supplementation treatment forms (OR 6·8 (95 % CI 2·7, 17), *P*<0·001) significantly increased the odds of having sufficient serum 25(OH)D concentration, whereas Ca with vitamin D and multivitamins were not significant determinates when compared with the non-supplemented group. Further, compared with the 0–9·9 μg/d group, all supplementation dose range categories significantly increased the odds of having sufficient serum concentrations 20–49·9 μg/d: (OR 4·2 (95 % CI 1·6, 10·6), *P*<0·03); 50–99·9 μg/d: (OR 10·8 (95 % CI 4, 30·6), *P*<0·001); >100 μg/d: (OR 14·7 (95 % CI 3·2, 68·5), *P*<0·001), except for the 10–19·9 μg/d group (OR 2·4 (95 % CI 0·9, 6·3), *P*=0·11).

## Discussion

With vitamin D’s role in the regulation of immunity, along with its potential to reduce the risk of viral infections, correcting insufficient serum 25(OH)D concentrations in older adults, especially those living in LTC communities, has never been more important than during the COVID-19 pandemic^(6,7)^. Our study adds to the LTC literature because it suggests that supplementation with vitamin D_2_or_3_ alone or a combination of therapies (including vitamin D, Ca with vitamin D and/or a multivitamin) may lead to a better ability to raise serum 25(OH)D to sufficient concentrations compared with a multivitamin or Ca with vitamin D supplement alone. Further, it also suggests that at least 37·5 μg/d of vitamin D is needed to maintain or treat insufficient serum 25(OH)D concentrations in older adults living in LTC.

The majority of national and international health organisations agree that individuals living in LTC communities should receive a vitamin D supplement; however, recommendations on the dose rate required and target serum 25(OH)D concentration remain controversial^(12,13,42)^. The wide variability in 25(OH)D assays has been cited as a limiting factor preventing consensus of serum cut-off points^(43)^. This study adds to the literature because it used 25(OH)D assays that were standardised and traceable to the gold standard 25(OH) vitamin D RMP from Ghent University (Ghent, Belgium)^(35)^. Further, we analysed these results in light of current Endocrine Society cut-off values for sufficiency/insufficiency; however, serum 25(OH)D concentration cut-off points used to define deficient, insufficient and sufficient remain controversial and highly debated among leading health organisations, specifically between The Endocrine Society’s 2012 and National Academy of Medicine’s 2011 guidelines^(43)^. It should be noted that the target audience on which the guidelines are based is a contributing factor to the controversy. The Endocrine Society’s guidelines are designed for clinical practice, while the National Academy of Medicine’s are designed for overall public health targeted at healthy non-diseased populations^(43)^.

Our results suggest that less than half of LTC residents had sufficient serum 25(OH)D concentration of ≥75 nmol/l. These results are similar to a study conducted in Pittsburgh, Pennsylvania showing that 52% of LTC residents have sufficient serum concentrations^(16)^. The number of LTC residents who received at least 5 μg/d of vitamin D supplementation in the current study (62%) was higher than expected based upon prior evidence suggesting ranges of 10–50%; however, 42% of those supplemented failed to reach sufficient serum 25(OH)D concentrations^(17,24,44)^. These data indicate that despite the higher-than-expected prevalence of supplementation, the prescribed supplementation dose rates were possibly too low, not treated for a long enough duration, or serum 25(OH)D concentrations were very low at the start of supplementation. These results are supported by multiple studies, including a 2015 randomised control trial of vitamin D-deficient LTC residents that showed supplementation of 20 μg/d took at least 12 weeks of supplementation for serum 25(OH)D concentrations to reach a steady state, and 60% of those never achieve the target serum 25(OH)D concentration of ≥75 nmol/l^(16,22,37)^. In the current study, all supplementation dose ranges were successful at reaching the target serum 25(OH)D concentrations of 75 nmol/l, expect dose ranges of <20 μg/d in at least some residents.

As expected, this study showed that as supplementation dose rate increases, serum 25(OH)D concentration also increases. Based on our predictive regression equation, we find that serum 25(OH)D concentrations increase approximately 8·0 nmol/l (3·0 ng/ml) for every 25 μg of vitamin D. A meta-analysis of sixteen randomised control trials of free-living adults by Black *et al.* also developed a predictive equation, and determined serum 25(OH)D concentrations increased 1·2 nmol/l for every 1 μg of ingested fortified foods^(45)^. Black *et al.* equation predicts a greater increase in serum concentration per μg supplemented than our study; however, the author acknowledges that the results should be interpreted with caution due to a high level of heterogeneity across the studies (i.e. environmental and methodology variability, population and age differences, range of assays used to measure 25(OH)D, daily doses of vitamin D and food sources). On the other hand, a study with a similar design as ours by Singh *et al.* concluded that a 5·0 nmol/l (2·0 ng/ml) increase in serum 25(OH)D concentrations occurred for every 25 μg of vitamin D supplemented in older adults in LTC communities^(19)^. Nevertheless, our study’s predicted rise in serum concentration of 8 nmol/l for every 25 μg of vitamin D shows that more vitamin D supplementation is required in the LTC population and is a concern even if targeted serum concentrations were <75 nmol/l.

Using the regression equation developed in our study, the findings suggest 37·5 μg/d of vitamin D is needed to achieve sufficient serum 25(OH)D concentrations. Several studies have also found similar results, including a study by Bischoff-Ferrari *et al.* that determined a supplementation dose rate >25 μg/d is required for older adults in LTC to achieve serum 25(OH)D concentration of ≥75 nmol/l^(20)^. Further, Kotlarcrzy *et al.* concluded that supplementation of 20 μg/d (800 IU)/ in LTC residents brought serum 25(OH)D concentrations to the National Academy of Medicine serum recommendation of ≥50 nmol/l, but these rates were not able to reach ≥75 nmol/l^(16)^. Thus, our results support the current recommendations set by the U.S. Endocrine Society Clinical Practice Guidelines that 37·5–50 μg/d (1500–2000 IU) of vitamin D is needed to reach and maintain 25(OH)D≥75 nmol/l^(12)^.

Our results should be interpreted in light of several study strengths and limitations. With regard to strengths, we were able to identify and control for medications metabolised by the cytochrome P450 25-hydroxylase enzyme, which has the potential to affect vitamin D status or alter supplementation effectiveness. In this study, 52% of participants received at least one medication with a drug–vitamin D interaction. Further, we were able to estimate daily vitamin D content provided in meals. Suominen *et al.* similarly found the nutrient content of vitamin D served to residents in LTC to be 5 μg/d with an estimated consumption of <2·5 μg/d^(46)^. Several study limitations also exist. Due to limitations of the retrospective study design and because of our desire to understand current LTC practices, we were unable to standardise food intake and physical activity (particularly outdoor activity reflective of direct sun exposure). However, it should be noted that it is a common practice by dietary supplement manufacturers to add ingredients greater than the label-claim (overage amount) to account for shelf life and losses during processing. These overages were not estimated in this study. With vitamin D overage amounts estimated to be up to ˜40%^(47)^, this could have resulted in an underestimate of vitamin D intake from supplementation and its effect on serum concentrations. Further, due to the available information in the medical record, we also were unable to determine total supplement duration and utilisation of loading doses. Loading doses are often prescribed to quicken return to a steady state, which often is not reached until 3–4 months of supplementation^(21,48,49)^. Compliance with supplementation was also not available; however, individuals living in LTC are reported to be 96% compliant when taking medications^(50)^. Nevertheless, because this study examines commonly used supplementation patterns in LTC residents, it does have high generalisability.

## Conclusion

Our results add to the growing need for health organisations to agree on serum cut-off concentrations used to define vitamin D status and provided clear clinical practice guidelines on dose rates and target serum 25(OH)D concentration for practitioners. We find that the current dietary reference intake of 20 μg/d may be too low to reach and maintain sufficient serum concentrations in older LTC residents. Our results support the U.S. Endocrine Society Clinical Practice Guidelines, which recommends 37·5–50 μg/d (1500–2000 IU) to maintain serum 25 (OH)D concentrations above 75 nmol/l^(12)^, but adds to this by suggesting that supplementation with either vitamin D_2or3_ alone or a combination vitamin D supplementation may be needed to achieve sufficient serum 25(OH)D concentration in patients living in LTC communities. Until a consensus is reached, individuals in LTC may continue to receive supplementation dosages that are ineffective at reaching sufficient serum 25(OH)D concentrations (>75 nmol/l), thus are potentially increasing the risk for adverse outcomes associated with non-skeletal health conditions^(3)^. These results have immediate implication and can be used by practitioners (physicians, dietitians and nurses) to maintain or achieve sufficient serum 25(OH)D concentrations in LTC older adults and potentially reduce COVID-19 infection rates, progression and disease severity.

## References

[1] Holick MF (2007) Vitamin D deficiency. N Engl J Med 357, 266–281. 17634462 10.1056/NEJMra070553

[2] Grant WB (2006) Epidemiology of disease risks in relation to vitamin D insufficiency. Prog Biophys Mol Biol 92, 65–79. 16546242 10.1016/j.pbiomolbio.2006.02.013

[3] Wimalawansa SJ (2018) Non-musculoskeletal benefits of vitamin D. J Steroid Biochem Mol Biol 175, 60–81. 27662817 10.1016/j.jsbmb.2016.09.016

[4] Nair R & Maseeh A (2012) Vitamin D: the “sunshine” vitamin. J Pharmacol Pharmacother 3, 118–126. 22629085 10.4103/0976-500X.95506 PMC3356951

[5] Ali N (2020) Role of vitamin D in preventing of COVID-19 infection, progression and severity. J Infect Public Health 13, 1373–1380. 32605780 10.1016/j.jiph.2020.06.021 PMC7305922

[6] Grant WB , Lahore H , McDonnell SL et al. (2020) Evidence that Vitamin D supplementation could reduce risk of influenza and COVID-19 infections and deaths. Nutrients 12, 988. 32492787 10.3390/nu12061620 PMC7352449

[7] Bilezikian JP , Bikle D , Hewison M et al. (2020) Mechanisms in endocrinology: vitamin D and COVID-19. Eur J Endocrinol 183, R133–R147. 32755992 10.1530/EJE-20-0665 PMC9494342

[8] van Schoor NM & Lips P (2011) Worldwide vitamin D status. Best Pract Res Clin Endocrinol Metab 25, 671–680. 21872807 10.1016/j.beem.2011.06.007

[9] Diekmann R , Winning K , Bauer JM et al. (2013) Vitamin D status and physical function in nursing home residents: a 1-year observational study. Z Gerontol Geriatr 46, 403–409. 23780630 10.1007/s00391-013-0507-7

[10] Center for Disease Control and Prevention (2020) COVID-19; Older Adults. https://www.cdc.gov/coronavirus/2019-ncov/need-extra-precautions/older-adults.html (accessed January 2021).

[11] Center for Disease Control and Prevention (2021) Morbidity and Mortality Weekly Report: Rates of COVID-19 among residents and staff in nursing homes. https://www.cdc.gov/mmwr/volumes/70/wr/mm7002e2.htm (accessed January 2021).

[12] Holick MF , Binkley NC , Bischoff-Ferrari HA et al. (2011) Evaluation, treatment, and prevention of vitamin D deficiency: an Endocrine Society clinical practice guideline. J Clin Endocrinol Metab 96, 1911–1930. 21646368 10.1210/jc.2011-0385

[13] Pilz S , Zittermann A , Trummer C et al. (2019) Vitamin D testing and treatment: a narrative review of current evidence. Endocr Connect 8, R27–R43. 30650061 10.1530/EC-18-0432 PMC6365669

[14] Rosen CJ , Abrams SA , Aloia JF et al. (2012) IOM committee members respond to Endocrine Society vitamin D guideline. J Clin Endocrinol Metab 97, 1146–1152. 22442278 10.1210/jc.2011-2218 PMC5393439

[15] Ross AC , Taylor CL , Yaktine AL et al. (2010) Dietary Reference Intakes for Calcium and Vitamin D. Washington, DC, USA: National Acaemic Press. 21796828

[16] Kotlarczyk MP , Perera S , Ferchak MA et al. (2017) Vitamin D deficiency is associated with functional decline and falls in frail elderly women despite supplementation. Osteoporos Int 28, 1347–1353. 27975302 10.1007/s00198-016-3877-z PMC6020826

[17] Rolland Y , de Souto Barreto P , Abellan Van Kan G et al. (2013) Vitamin D supplementation in older adults: searching for specific guidelines in nursing homes. J Nutr Health Aging 17, 402–412.2353866710.1007/s12603-013-0007-x

[18] Holick MF (2011) Vitamin D deficiency in 2010: health benefits of vitamin D and sunlight: a D-bate. Nat Rev Endocrinol 7, 73–75. 21263437 10.1038/nrendo.2010.234

[19] Singh G & Bonham AJ (2014) A predictive equation to guide vitamin D replacement dose in patients. J Am Board Fam Med 27, 495–509. 25002004 10.3122/jabfm.2014.04.130306

[20] Bischoff-Ferrari HA , Giovannucci E , Willett WC et al. (2006) Estimation of optimal serum concentrations of 25-hydroxyvitamin D for multiple health outcomes. Am J Clin Nutr 84, 18–28. 16825677 10.1093/ajcn/84.1.18

[21] Schwartz JB , Kane L & Bikle D (2016) Response of Vitamin D concentration to vitamin D_3_ administration in older adults without sun exposure: a randomized double-blind trial. J Am Geriatr Soc 64, 65–72. 26782853 10.1111/jgs.13774 PMC4724876

[22] Chel V , Wijnhoven HA , Smit JH et al. (2008) Efficacy of different doses and time intervals of oral vitamin D supplementation with or without calcium in elderly nursing home residents. Osteoporos Int 19, 663–671. 17874029 10.1007/s00198-007-0465-2 PMC2277446

[23] Rolland Y , Abellan van Kan G , Hermabessiere S et al. (2009) Descriptive study of nursing home residents from the REHPA network. J Nutr Health Aging 13, 679–683.1965755010.1007/s12603-009-0197-4

[24] Buckinx F , Reginster JY , Cavalier E et al. (2016) Determinants of vitamin D supplementation prescription in nursing homes: a survey among general practitioners. Osteoporos Int 27, 881–886. 26733374 10.1007/s00198-015-3469-3

[25] Center for Medicare and Medicaid Services (2019) Medical Necessity for Vitamin Assay D Testing. https://www.cms.gov/Research-Statistics-Data-and-Systems/Monitoring-Programs/Medicare-FFS-Compliance-Programs/Recovery-Audit-Program/Approved-RAC-Topics-Items/0143-Medical-Necessity-for-Vitamin-D-Assay-Testing-CPT-82306-CPT-82652 (accessed April 2019).

[26] Karanicolas PJ , Farrokhyar F & Bhandari M (2010) Practical tips for surgical research: blinding: who, what, when, why, how? Can J Surg 53, 345–348. 20858381 PMC2947122

[27] Li T , Vedula SS , Hadar N et al. (2015) Innovations in data collection, management, and archiving for systematic reviews. Ann Intern Med 162, 287–294. 25686168 10.7326/M14-1603

[28] U.S. Census Bureau (2018) 2013–2017 ACS 5-year Estimates. https://www.census.gov/programs-surveys/acs/technical-documentation/table-and-geography-changes/2017/5-year.html (accessed April 2019).

[29] Center for Disease Control and Prevention (2020) About Adult BMI. https://www.cdc.gov/healthyweight/assessing/bmi/adult_bmi/index.html (accessed January 2018).

[30] Food and Drug Administration (2018) USP Therapeutic Categories Model Guidelines. https://www.fda.gov/regulatory-information/fdaaa-implementation-chart/usp-therapeutic-categories-model-guidelines (accessed April 2018).

[31] Robien K , Oppeneer SJ , Kelly JA et al. (2013) Drug-vitamin D interactions: a systematic review of the literature. Nutr Clin Pract 28, 194–208. 23307906 10.1177/0884533612467824 PMC5623087

[32] U.S. Department of Agriculture (2018) USDA Food Composition Databases. https://fdc.nal.usda.gov (accessed June 2018).

[33] Lips P , van Schoor NM & de Jongh RT (2014) Diet, sun, and lifestyle as determinants of vitamin D status. Ann N Y Acad Sci 1317, 92–98. 24814938 10.1111/nyas.12443

[34] Sliney DH & Wengraitis S (2006) Is a differentiated advice by season and region necessary? Prog Biophys Mol Biol 92, 150–160. 16682072 10.1016/j.pbiomolbio.2006.02.007

[35] Beckman Coulter, Inc (2020) Instructions for Use: Access 25(OH)D Vitamin D Total 25(OH)D vitamin D. In. B29609 D. https://www.beckmancoulter.com/download/file/phxB29609F-EN_US/B29609F?type=pdf (accessed October 2020).

[36] Madenci Ö , Orçun A , Yildiz Z et al. (2017) Evaluation of new Beckman Coulter 25(OH) Vitamin D assay and potential improvement of clinical interpretation. Biochem Med (Zagreb) 27, 332–341. 28694724 10.11613/BM.2017.036 PMC5493169

[37] Wijnen H , Salemink D , Roovers L et al. (2015) Vitamin D supplementation in nursing home patients: randomized controlled trial of standard daily dose versus individualized loading dose regimen. Drugs Aging 32, 371–378. 25899914 10.1007/s40266-015-0259-8

[38] Institute of Medicine (2011) Dietary Reference Intake for Calcium and Vitamin D. Washington, DC: The National Academies Press. 21796828

[39] Gallagher JC , Sai A , Templin T 2nd et al. (2012) Dose response to vitamin D supplementation in postmenopausal women: a randomized trial. Ann Intern Med 156, 425–437. 22431675 10.7326/0003-4819-156-6-201203200-00005

[40] Gallagher JC , Peacock M , Yalamanchili V et al. (2013) Effects of vitamin D supplementation in older African American women. J Clin Endocrinol Metab 98, 1137–1146. 23386641 10.1210/jc.2012-3106 PMC3590472

[41] American Geriatrics Society Workshop on Vitamin D Supplementation for Older Adults (2014) Recommendations abstracted from the American geriatrics society consensus statement on vitamin D for prevention of falls and their consequences. J Am Geriatr Soc 62, 147–152. 24350602 10.1111/jgs.12631

[42] Fuleihan Gel H , Bouillon R , Clarke B et al. (2015) Serum 25-Hydroxyvitamin D Levels: variability, knowledge gaps, and the concept of a desirable range. J Bone Miner Res 30, 1119–1133. 25952470 10.1002/jbmr.2536

[43] Sempos CT & Binkley N (2020) 25-Hydroxyvitamin D assay standardisation and vitamin D guidelines paralysis. Public Health Nutr 23, 1153–1164. 32301688 10.1017/S1368980019005251 PMC7167380

[44] Bruyere O , Cavalier E , Souberbielle JC et al. (2014) Effects of vitamin D in the elderly population: current status and perspectives. Arch Public Health 72, 32. 25279143 10.1186/2049-3258-72-32 PMC4181706

[45] Black LJ , Seamans KM , Cashman KD et al. (2012) An updated systematic review and meta-analysis of the efficacy of vitamin D food fortification. J Nutr 142, 1102–1108. 22513988 10.3945/jn.112.158014

[46] Suominen MH , Hosia-Randell HM , Muurinen S et al. (2007) Vitamin D and calcium supplementation among aged residents in nursing homes. J Nutr Health Aging 11, 433–437. 17657365

[47] Andrews KW , Gusev PA , McNeal M et al. (2018) Dietary supplement ingredient database (DSID) and the application of analytically based estimates of ingredient amount to intake calculations. J Nutr 148, Suppl 2, 1413s–1421s. 31505677 10.1093/jn/nxy092 PMC6857613

[48] Heaney RP , Davies KM , Chen TC et al. (2003) Human serum 25-hydroxycholecalciferol response to extended oral dosing with cholecalciferol. Am J Clin Nutr 77, 204–210. 12499343 10.1093/ajcn/77.1.204

[49] Jones KS , Assar S , Harnpanich D et al. (2014) 25(OH)D2 half-life is shorter than 25(OH)D3 half-life and is influenced by DBP concentration and genotype. J Clin Endocrinol Metab 99, 3373–3381. 24885631 10.1210/jc.2014-1714 PMC4207933

[50] Veleva BI , Chel VG & Achterberg WP (2014) Efficacy of daily 800 IU vitamin D supplementation in reaching vitamin D sufficiency in nursing home residents: cross-sectional patient file study. BMC Geriatr 14, 103. 25238786 10.1186/1471-2318-14-103 PMC4246443

